# Hedgehog fuels gut regeneration

**DOI:** 10.18632/oncotarget.4929

**Published:** 2015-07-21

**Authors:** Aiguo Tian, Jin Jiang

**Affiliations:** Department of Developmental Biology, UT Southwestern Medical School at Dallas, TX, USA

**Keywords:** Chromosome Section, Hedgehog, midgut, ISC, regeneration

The intestinal epithelia rely on resident stem cells to undergo homeostatic growth as well as tissue repair upon injury. In response to insults elicited by pathological bacterial infection or tissue damaging chemicals such as dextran sodium sulfate (DSS), intestinal stem cells (ISCs) speed up their proliferation and differentiation to effectively replenish lost cells. Despite numerous studies, the regulatory mechanisms underlying the control of stem cell activity during homeostasis and regeneration still remain poorly understood.

The Hedgehog (Hh) pathway governs animal development in species ranging from fruit fly to human. In additional to their role as morphogen to control cell differentiation and pattern formation in embryonic development, the secreted Hh glycoproteins also function as mitogen to regulate cell proliferation in adult tissue homeostasis and tumorigenesis [1]. Hh signaling has been implicated in tissue repair in organs including lung, prostate, pancreas, liver, bladder, skin, and bone. With few exceptions, the precise locations where Hh signaling acts and the downstream genes mediating the effect of Hh signaling have remained largely unknown. *Drosophila* adult midgut provides a powerful system to address these problems because sophisticated genetic tools are available to allow the manipulation of gene function in any cell types within the intestinal epithelia as well as in surrounding tissues. Our recent study, which was published in *The Journal of Cell Biology*, established a critical role of Hh signaling in the regulation of regenerative stem cell proliferation in *Drosophila* adult midguts [2].

Similar to mammalian intestines, *Drosophila* midguts constantly turn over and are replaced by new cells derived from ISCs localized at the base of the epithelia. ISCs divide to produce themselves and enteroblasts (EBs) that subsequently differentiate into more specialized cell types. Previous studies revealed that ISC proliferation was elevated upon intestinal injury caused by DSS feeding [3]. Here we found that DSS increased the production of Hh ligand in multiple cell types including precursor cells (ISCs plus EBs) and enterocytes but Hh pathway activity was only elevated in precursor cells. Depleting Hh from the precursor cells but not from the enterocytes blocked DSS-stimulated ISC proliferation, suggesting that local Hh production is critical for stimulating ISC proliferation. Surprisingly, we found that blocking Hh pathway activity in EBs but not in ISCs affected DSS-induced ISC proliferation, suggesting that Hh signaling didn't drive ISC proliferation by acting directly on the stem cells themselves but rather by acting on the neighboring EBs. In corroborating with this non-cell-autonomous role of Hh signaling, activation of the Hh pathway in EBs either by expressing a constitutively active form of the pathway effector Ci or by removing a pathway inhibitor Ptc drove ISC proliferation. By contrast, activation of Hh signaling in stem cells failed to drive ISC proliferation. The non-cell-autonomous function of Hh signaling we observed here is reminiscent of what has been observed in certain cancers where Hh derived from cancer cells acts on surrounding stromal cells to indirectly promote cancer cell proliferation [4].

In searching for the effectors that mediate the Hh function in midguts, we found that Hh signaling in EBs upregulated several cytokines and mitogens, most notably, the JAK-STAT pathway ligand Upd2. Indeed, inaction of Upd2 in EBs blocked ISC proliferation induced by either DSS feeding or ectopic Hh signaling. Taken together, our study suggests that DSS stimulates Hh signaling in EBs where it increases the production of Upd2, which in turn activates the JAK-STAT pathway in stem cells to fuel their proliferation. Interestingly, a recent study revealed a similar Hh-JAK-STAT signaling axis that drives tumorigenesis in mouse skin [5], suggesting that the signaling network we uncovered here is likely to be widely utilized to control stem cell proliferation.

How Hh production is upregulated upon injury remains poorly understood but our study revealed that DSS increased Hh expression in part through the JNK pathway although the precise mechanism remains to be further explored. Another unresolved issue is how Hh signaling regulates the production of Upd2. Hh could directly regulate the transcription of *upd2* or indirectly through an intermediate pathway. In this regard, it is interesting to note that the Hippo (Hpo) pathway is also involved in DSS-stimulated ISC proliferation [6], raising an interesting possibility that Hh and Hpo pathways may act in concert to regulate ISC proliferation. Indeed, our unpublished data revealed that activation of the Hpo pathway effector Yki in EBs also upregulated Upd2 and that inactivation of Yki inhibited Hh stimulated ISC proliferation. Future study will address the precise mechanism by which Hh and Hpo signaling pathways intersect to control stem cell proliferation in *Drosophila* midguts and perhaps also in other settings.
